# Addressing cognitive impairment in peritoneal dialysis: a systematic review and meta-analysis of prevalence, risk factors, and outcomes

**DOI:** 10.1093/ckj/sfae312

**Published:** 2024-10-15

**Authors:** Noppawit Aiumtrakul, Charat Thongprayoon, Pitchaporn Yingchoncharoen, Chalothorn Wannaphut, Wannasit Wathanavasin, Supawadee Suppadungsuk, Pajaree Krisanapan, Wisit Cheungpasitporn

**Affiliations:** Department of Medicine, John A. Burns School of Medicine, University of Hawaii, Honolulu, HI, USA; Division of Nephrology and Hypertension, Department of Medicine, Mayo Clinic, Rochester, MN, USA; Department of Medicine, Texas Tech University Health Sciences Center, Lubbock, TX, USA; Department of Medicine, John A. Burns School of Medicine, University of Hawaii, Honolulu, HI, USA; Division of Nephrology and Hypertension, Department of Medicine, Mayo Clinic, Rochester, MN, USA; Nephrology Unit, Department of Medicine, Charoenkrung Pracharak Hospital, Bangkok Metropolitan Administration, Bangkok, Thailand; Division of Nephrology and Hypertension, Department of Medicine, Mayo Clinic, Rochester, MN, USA; Chakri Naruebodindra Medical Institute, Faculty of Medicine Ramathibodi Hospital, Mahidol University, Samut Prakan, Thailand; Division of Nephrology and Hypertension, Department of Medicine, Mayo Clinic, Rochester, MN, USA; Department of Internal Medicine, Faculty of Medicine, Thammasat University, Pathum Thani, Thailand; Division of Nephrology and Hypertension, Department of Medicine, Mayo Clinic, Rochester, MN, USA

**Keywords:** cognitive impairment, dementia, end-stage renal disease, peritoneal dialysis

## Abstract

**Background:**

Cognitive impairment (CI) is a critical complication in peritoneal dialysis (PD) patients, associated with decreased quality of life and increased hospitalization. Despite its significant impact, the prevalence, risk factors, and consequences of CI in PD patients are not well understood. We aimed to determine the prevalence, risk factors, and outcomes of CI in PD patients.

**Methods:**

We performed systematic reviews in OVID Medline, Embase, and Cochrane databases until February 2024 to identify cross-sectional and cohort studies on prevalence of CI (identified by cognitive assessment scales) in PD patients. The Newcastle–Ottawa Scale was used to assess risk of bias. A pooled meta-analysis of CI prevalence in PD and a subgroup analysis comparing the risk of CI between PD and non-PD settings were performed using a random-effects model.

**Results:**

A total of 19 studies were identified, involving 2882 PD patients. The pooled prevalence of CI in PD patients was 47.7% (95%CI: 35.8–59.9%). CI in patients undergoing PD appears to be associated with older age, female gender, lower levels of education, and is linked to higher rates of hospitalization and peritonitis, compared to those without CI. However, it is not associated with increased mortality. Compared to hemodialysis, PD showed a trend toward a lower risk of CI (OR 0.64, 95%CI 0.39–1.03; *P *= .068).

**Conclusion:**

CI is highly prevalent and associated with several adverse clinical outcomes in PD patients. These findings could contribute to facilitate the development of screening and early intervention strategies to reduce the burden of disease in this population.

## INTRODUCTION

The global prevalence of end-stage renal disease (ESRD) has increased over the past few decades, largely due to aging populations, rising incidence of diabetes mellitus (DM) and hypertension, and improved survival rates of patients with chronic kidney disease (CKD) [1, 1]. ESRD patients are managed through renal replacement therapy, predominantly using either peritoneal dialysis (PD) or hemodialysis based on various factors such as comorbidities, socioeconomic status, patient or physician preference, and public policy [2, 3]. In the USA, data from 2020 revealed that 16 528 patients initiated PD, representing 12.7% of individuals with incident ESRD, more than doubling the percentage observed in 2008 [4].

Cognitive impairment (CI) is common among patients with ESRD, with a 2- to 7-fold higher prevalence than the general population [5–7]. CI among ESRD patients is important due to its association with poor engagement with healthcare, worsened physical and mental health, and increased morbidity and mortality. Furthermore, given that PD is a home-based therapy, CI in PD patients may increase the risk of procedural errors leading to peritonitis [8, 9]. Wang *et al.* revealed a higher prevalence of CI among patients receiving PD compared to those not requiring dialysis [10].

Despite its significant impact on patient outcomes, the prevalence, risk factors, and consequences of CI in PD patients are not well understood. This systematic review and meta-analysis aimed to determine the prevalence, risk factors, and outcomes of CI in PD patients, which is crucial for early recognition, prevention, and management.

## MATERIALS AND METHODS

### Search strategy and literature review

This study was performed according to the Preferred Reporting Items for Systematic Reviews and Meta-Analysis guideline [11] (Supplementary material 2) and the Strengthening the Reporting of Observational Studies in Epidemiology [12]. The study protocol was registered in the International Prospective Register of Systematic Reviews (CRD42024526057). A systematic literature search of EMBASE (1988 to February 2024), OVID MEDLINE (1946 to February 2024), and the Cochrane Central Register of Controlled Trials (database inception to January 2024) and Cochrane Database of Systematic Reviews (2004 to February 2024) was performed to assess the prevalence, risk factors, and outcomes of CI in PD. The search terms included (“peritoneal dialysis” AND “cognitive dysfunction” OR “cognitive impairment” OR “cognitive disorder” OR “cognitive decline” OR “mental deterioration” OR “dementia”). The searched article was limited to studies in humans that were published in English.

### Inclusion and exclusion criteria

**Figure 1:**
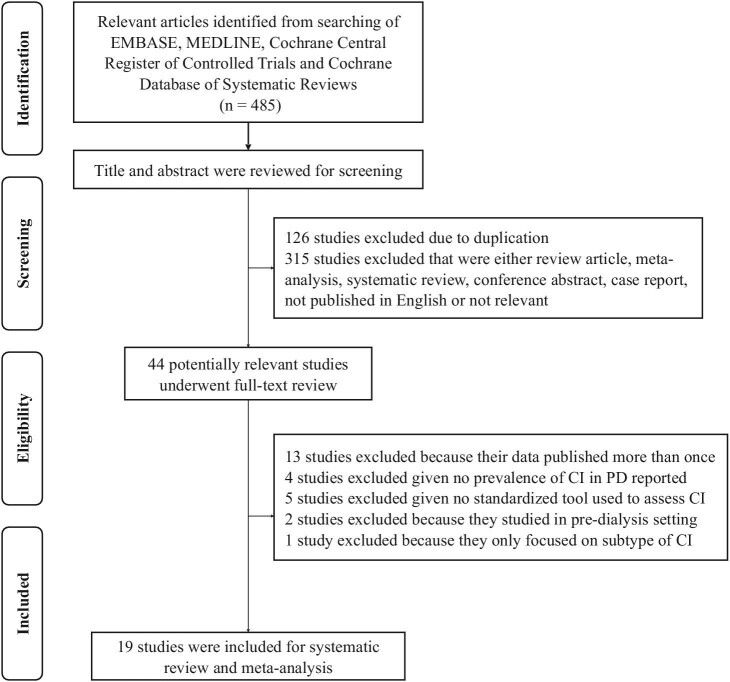
Flowchart of search methodology.

The inclusion criteria were as follows: (i) original research articles in a cross-sectional, prospective, or retrospective design; (ii) participants must be adult patients (18 years or older) undergoing PD; (iii) the study must utilize standardized cognitive tools to diagnose CI; and (iv) the study must report the prevalence of CI in PD or provide adequate information to calculate the prevalence of CI in PD. The exclusion criteria were as follows: (i) data had been published more than once; and (ii) a review article, meta-analysis, systematic review, conference abstract, or case report (Fig. 1). The relevant literature was systematically searched and reviewed by two investigators (N.A. and P.Y.) independently. If any of the findings had inconsistent results, it would be concluded by consensus.

### Data extraction

The following information was extracted from each relevant article: (i) authors; (ii) year of publication; (iii) country of origin; (iv) number of dialysis center(s) involved; (v) sample size; (vi) mean age; (vii) sex; (viii) BMI; (ix) mode of PD; (x) dialysis vintage; (xi) level of education; (xii) comorbidities; (xiii) prevalence of CI; (xiv) cognitive exam; (xv) reported risk factors of CI; (xvi) clinical outcomes, including infection, hospitalization and mortality; and (xvii) control groups of hemodialysis, renal-transplantation, and non-dialysis CKD patients.

### Assessment of quality and risk of bias

The risk of bias among the included studies was evaluated using the Newcastle–Ottawa Scale (NOS). The NOS consists of a set of questions aimed at assessing the selection of subjects, the comparability of each study, and the outcomes. We utilized the adapted NOS for cross-sectional studies. Cross-sectional studies were categorized based on their risk of bias: those scoring 7 or 6 points were categorized as low risk, while those scoring 5 points were considered to have a medium risk. Studies scoring 4 points or less were classified as high risk of bias. For cohort studies, studies with higher scores (8–9 points) were considered to have a low risk of bias, while those with lower scores (5 points or less) were deemed to have a high risk of bias. Studies scoring between 6 and 7 points were classified as having a medium risk of bias [13, 14]. N.A. and P.Y. independently evaluated the quality of each included article using the specified assessment criteria. Any disagreements in the assessment were resolved through discussion with W.W.

### Statistical analysis

Pooled estimate of the prevalence of CI was calculated using the random-effects model. To evaluate heterogeneity among the studies, we applied Cochran's *Q* test and the *I*² statistic. The *I*² statistic provides a degree of heterogeneity, with values ranging from 0% to 100%. Values between 0% and 25% indicate insignificant heterogeneity, while values between 26% and 50% suggest low heterogeneity. Levels between 51% and 75% indicate moderate heterogeneity, and values from 76% to 100% suggest high heterogeneity. Subgroup analysis was used to assess variations in prevalence estimates across different types of cognitive exams, study characteristics and various study populations, including non-CKD, non-dialysis-dependent CKD, and HD. Odd ratios (ORs) were utilized as measures of effect estimates across the included studies. Publication bias was qualitatively and quantitatively assessed using visual inspection of funnel plots and the Egger's test, respectively. Meta-regression analysis was conducted to explore trends of the prevalence of CI over time, dialysis vintage, and age. These analyses were carried out using the Comprehensive Meta-Analysis v.3.3 software (Biostat Inc., Englewood, NJ, USA) and the bubble plot for meta-regression analysis was generated using STATA^®^ (Statacorp. Version 18. College Station, TX, USA).

## RESULTS

A total of 485 potentially relevant studies were identified. After screening titles and abstracts, we excluded 126 duplicates and 315 studies with irrelevant information, written in a language other than English, or meeting the exclusion criteria (review articles, meta-analyses, systematic reviews, conference abstracts, or case reports). Then, 44 related studies were left for comprehensive review. We additionally excluded 13 studies that reported the same data more than once, four studies that did not report prevalence of CI in PD or did not provide adequate data to calculate prevalence of CI in PD, five studies that did not utilize standardized cognitive tools to assess CI, two studies conducted in pre-dialysis setting, and one study that solely focused on subtype of CI. This resulted in a final selection of 19 eligible studies [5–10, 15–27], involving 2882 PD patients.

### Baseline characteristics

Among PD patients, 52.38% of subjects were male. The weighted mean age was 55.02 ± 9.42 years, the weighted mean BMI was 22.75 ± 3.50 kg/m^2^, the weighted mean dialysis vintage was 28.93 ± 29.79 months. Nine out of 19 studies reported modes of PD, which either Continuous Ambulatory PD (CAPD) or Automated PD (APD). Of PD patients, 85.77% were CAPD, while 14.23% were APD. 16 of 19 studies reported levels of education, the weighted mean years of education was 8.75 ± 3.56 years with 64.61% of PD subjects had education equal to or less than 12 years or high school level.

Hypertension was the most common comorbidity, ranging from 81% to 96.3% across studies. The prevalence of DM varied between 19.7% and 55% The prevalence of coronary artery disease, cerebrovascular accident and peripheral vascular disease were 11%–60%, 3.7%–85.2%, and 1.2%–32%, respectively. Of PD patients, 26.7% had chronic lung diseases, 5%–8% had underlying malignancy, while 1.2%–18.5% had psychiatric illness, including depression. A study by Wang *et al.*, reported 83.53% of PD patients had pre-existing chronic glomerulonephritis [10] (Tables 1 and 2).

**Table 1:** Cross-sectional studies on the prevalence of CI in PD patients with/without control groups.

Study [authors, year of publication, country, center(s)]	Sample size (*n*)	Mean age (years)	Sex ratio (M/F)	BMI (kg/m^2^)	Mode of PD	Dialysis vintage (months)	Education ≤12 years, equal to or less than high school (%) or reported as mean ± SD	Comorbidities (%)	Prevalence of CI (%)	Cognitive exam	Risk factors of CI
Li *et al.* [5], 2004, Hong Kong, Single center	81	71.6 ± 4.2	41/40	N/A	CAPD (100%, *n* = 81)	36.4 ± 29.3	3.77 ± 3.72	DM (51.9%) HTN (96.3%) IHD (42%) CVA (18.5%) PVD (1.2%) CAD (27.2%) Psychiatric illness (1.2%)	32.1% (*n* = 26)	Cantonese MMSE ≤18/30 for illiterate patients, ≤20/30 for 1–2 years of schooling, and ≤22/30 for those with more than 2 years of schooling	CAPD: OR 7.7, 95% CI, 2.4–24.8; *P* < .001. Age: OR 1.1, 95% CI, 1.02–1.3; *P* = .018. Female gender: OR, 3.3; 95% CI, 1.1–9.9; *P* < .03 Years of education: OR 0.78, 95% CI, 0.65–0.94; *P* < .009.
	66 (healthy subjects)	71.77 ± 5.59	31/35	N/A	N/A	N/A	4.85 ± 4.19	N/A	7.6% (*n* = 5)		
Sithinamsuwan *et al.* [7], 2005, Thailand, Single center	30	55.7 ± 14.2	21/9	N/A	CAPD (100%, *n* = 30)	36.67 ± 30.62	11.13 ± 4.83	DM (33.3%) HTN (83.3 %) Depression (6.7 %)	3.3% (*n* = 1)	Thai MSE < 24/30	N/A
	60 (HD)	53.67 ± 15.84	33/27	N/A	N/A	68.48 ± 34.04	9.98 ± 4.67	DM (20.0%) HTN (95.0%) Depression (6.7%)	8.3% (*n* = 5)		
Kalirao *et al.* [15], 2011, United States, 2 PD units	51	57.5 ± 14.8	34/17	N/A	CAPD (15.69%, *n* = 8) APD (84.31%, *n* = 43)	23 ± 15.6	46% or 13.2 ± 2.4 years	DM (41.2%) CVA (11.8%)	74.5% (*n* = 38) Mild (7.8%, *n* = 4) Mod (33.3%, *n* = 17) Severe (33.3%, *n* = 17)	A 45-minute battery of 9 validated neuropsychological tests, including 3MS	PD as a risk of mod-severe CI: OR 2.58 (1.02–6.53)
	338 (HD)	71.2 ± 9.5	183/155	N/A	N/A	32.8 32.8	54.7% or 12.8 3.0 years	DM (46.8%) CVA (23.4%)	87.28% (*n* = 295) Mild (13.9%, *n* = 47) Mod (36.1%, *n* = 122) Severe (37.3%, *n* = 126)		HD as a risk of mod-severe CI: OR 3.16 (1.91–5.24)
	101 (non-CKD)	68.5 ± 9.6	44/57	N/A	N/A	N/A	32.7% or 14.3 3.0 years	DM (22.8%) CVA (7.9%)	52.48% (*n* = 53) Mild (12.87%, *n* = 13) Mod (26.73%, *n* = 27) Severe (12.87%, *n* = 13)		Non-CKD as a risk of mod-severe CI: OR 1.00 (ref) adjusted for age, sex, race, education, DM, and stroke
Jung *et al.* [6], 2013, Korea, Single center	27	52.4 ± 11.6	14/13	24.1 ± 4.3	N/A	31.2 ± 19.2	48.1%	DM (33.3%) CVD (33.3%) Smoking (22.2%) Depression (18.5%)	11.1% (*n* = 3)	K-MMSE ≤ 24	N/A
	29 (HD)	55.8 ± 8.7	13/16	22.4 ± 3.2	N/A	67.2 ± 56.4	48.3%	DM (41.4%) CVD (44.8%) Smoking (20.7%) Depression (51.7%)	24.1% (*n* = 7)		
Isshiki *et al.* [16], 2014, Japan, Single center	18	67.5 ± 6.9	12/6	N/A	N/A	29 (8–105)	N/A	DM (44.4%) HTN (94.4%) IHD (11.1%) Smoker (22.2%)	22.2% (*n* = 4) Mild (16.67%, *n* = 3) Mod (5.56%, *n* = 1)	MMSE ≤ 26	N/A
Lambert *et al.* [17], 2016, Australia, Single center	25	70 (63–81)	13/12	N/A	N/A	30 (18–48)	72%	Lung diseases (15.0%) CAD (40%) PAD (20%) DM (35%) CVA (10%) Cancer (5%)	48.0% (*n* = 12)	MoCA ≤ 24	N/A
	24 (pre-dialysis)	70 (63–76)	11/13	N/A	N/A	N/A	54.2%	N/A	16.7% (*n* = 4)		
	54 (HD)	72.0 (58–77)	36/18	N/A	N/A	4.25 (2–9)	63.0%	Lung diseases (26.7%) CAD (60%) PAD (57.8%) DM (51.1%) CVA (31.1%) Cancer (20.0%)	55.6% (*n* = 30)		
	52 (KT)	58.5 (49–66)	32/20	N/A	N/A	8.1 (4.1–14.3)	44.2%	Lung diseases (16.3%) CAD (26.5%) PAD (32.7%) DM (28.6%) CVA (16.3%) Cancer (34.7%)	19.2% (*n* = 10)		
Zheng *et al.* [18], 2017, China, Single center	72	56.2 ± 16.0	27/45	22.9 ± 3.6	N/A	41.2 ± 36.1	59.70%	CSVD; lacunar infarcts (38.9%), microbleeds (36.1%), WMHs (48.6%), and ICH (4.2%) DM (31.9%) HTN (90.3%) Smoking (16.7%)	86.6% (*n* = 58/67) by MoCA (25% by MMSE)	Chinese MoCA ≤ 26, if schooling <12 years, 1 point added Chinese MMSE ≤19 for illiterate subjects, ≤22 for education <7 years and ≤26 for education >7 years	Lacunar infarct is significantly related to cognitive decline *P *< .001
Salazar-Felix *et al.* [19], 2021, Mexico, Single center	71	43 ± 16	56/15	27 ± 4	APD (100%, *n* = 71)	17 (7–32)	84.51%	DM (34%) HTN (81%) Smoking (4%)	64.79% (*n* = 46) MoCA (100%), MMSE (7% of CI diagnosis)	MoCA ≤ 25, if schooling <12 years, 1 point added	Multiple linear regression analysis for predicting the MoCA in APD Education (years): *B* = .53, 95%CI 0.19–0.87, *P *= .003 Serum Na: *B* = .56, 95%CI 0.04–1.08, *P *= .03 Serum creatinine: B = .48, 95%CI 0.06–0.91, *P *= .03 Age (years): B = −.10, 95%CI −0.20–0.00, *P *= .05
	71 (controls)	42 ± 17	55/16	27 ± 4	N/A	N/A	83.10%	DM (34%) HTN (20%) Smoking (17%)	36.62% (*n* = 26) MoCA (100%), MMSE (4% of CI diagnosis)		N/A
Gamage *et al.* [20], 2022, Australia, Single center	149	60.8 ± 15.1	100/49	N/A	APD (97.2%, *n* = 145) CAPD (2.68%, *n* = 4)	17.03 ± 16.67	N/A	DM (48.3%) IHD (30.9%) CVA (85.2%)	36.9% (*n* = 55) ACE-R (100%), MMSE (9.1% of CI diagnosis)	ACE-R (cut-off point not defined) MMSE (cut-off point not defined)	Risk factors for lower ACE-R score included age, male, DM, depression, and PD vintage beyond 12 months. The protective factor included residual renal function
Wang *et al.* [10], 2022, China, Single center	85	N/A	46/39	N/A	N/A	N/A	N/A	CGN (83.53%)	90.6% (*n* = 78)	MoCA < 26	N/A
	88 (controls)	N/A	48/40	N/A	N/A	N/A	N/A	N/A	59.1% (*n* = 52)		N/A
Golenia *et al.* [21], 2023, Poland, Single center	18	50 ± 19	5/13	N/A	N/A	47.6 ± 37.9	14.5 ± 3.4	N/A	33.3% (*n* = 6)	ACE III test ≤88%	ACE III was significantly positively correlated with years of education (*r* = .78), *P *< .001
	15 (controls)	49 ± 20	3/12	N/A	N/A	N/A	14.1 ± 2.7	N/A	27% (*n* = 4)		ACE III was significantly positively correlated with years of education (*r* = .53), *P *= .04
Wu *et al.* [22], 2023, China, Single center	98	<55 y (32.65%), 55–64 y (43.88%), and 65 y (23.47%)	48/40	22.13 ± 3.32	CAPD (100%, *n* = 98)	29.89 ± 15.74	54.09%	DM (44.90%) HTN (87.86%) CVA (10.2%) CVD (29.59%)	69.39% (*n* = 68)	MoCA < 26	N/A

Abbreviation; 3MS, Modified Mini-Mental State Examination; TMT-B, Trail-Making Test B; HK-MoCA, Hong Kong Montreal Cognitive Assessment; d2-R, Test d2-Revision; ICD-9-CM, International Classification of Diseases, 9th revision, Clinical Modification; CSVD, cerebral small vessel diseases; WMH, abnormal brain white matter hyperintensity; ICH, intracerebral hemorrhage.

**Table 2:** Longitudinal studies on the prevalence of CI in PD with/without control groups.

Study	Follow-up time (months)	Sample size (*n*)	Mean age (years)	Sex ratio (M/F)	BMI (kg/m^2^)	Mode of PD	Dialysis vintage (months)	Education ​​<12 years, equal to or less than high school (%) or reported as mean ± SD	Comorbidities (%)	Prevalence of CI (%)	Cognitive exam	Risk factors of CI	Outcomes
Iyasere *et al.* [26], 2016, UK, Prospective, 3 outpatient clinics	12 (6–18)	25	72.8 ± 1.6	19/6	N/A	N/A	8 (5–32)	0.00%	DM (44%) IHD (40%) PVD (32%) LV dysfunction (16%) Malignancy (8%)	64.3% (*n* = 15)	MoCA < 26	MoCA executive score declined faster in HD compared to PD (coefficient −.12, 95%CI −0.23–−0.01; *P *= .037)	N/A
		41 (HD)	68.9 ± 1.3	29/12	N/A	N/A	35 (15.5–60)	38.9%	DM (46.3%) IHD (46.3%) PVD (17.1%) LV dysfunction (9.8%) Malignancy (7.3%)	63.6% (*n* = 26)			N/A
		36 (CKD)	72.5 ± 1.5	23/13	N/A	N/A	N/A	35.0%	DM (66.7%) IHD (50%) PVD (22.2%) LV dysfunction (13.9%) Malignancy (2.8%)	53.8 (*n* = 19)		MoCA scores declined faster in dialysis compared with CKD patients (coefficient .03, 95%CI 0.06 to 0.004; *P *= .025)	N/A
Neumann *et al.* [27], 2017, Germany, Prospective, 55 dialysis units	12	108	56 ± 14.7	71/37	N/A	N/A	14.9 ± 7.2	65.74% (*n* = 71)	CVA 6.5%	15% (*n* = 16)	TMT-B, Test d2-R, and subscale KDQOL-CF short form	N/A	N/A
		163 (HD)	57 ± 15	119/44	N/A	N/A	14.8 ± 5.7	82.2% (*n* = 134)	CVA 3.7%	29% (*n* = 48)			N/A
Zhang *et al.* [25], 2018, China, Prospective, 5 PD centers	24	458	51.6 ± 14.2	243/215	22.7 ± 3.5	N/A	25.1 (11.1–49.0)	47.6 % (*n* = 218)	DM (19.7%) CVA (17.5%)	19.7% (*n* = 90)	3MS TMT Subtests of RBANS	Multivariable Linear Regression Models on 3MS Score (B (SE), *P*) Age: *B* = −0.12 (0.05), *P *= .01* Depression score: *B*= −0.14 (0.07) *P *= .04* Levels of education: *B* = 2.86 (0.65), *P *< .001 Serum albumin: *B* = 0.64 (0.11), *P *< .001*	Risk of CI on: First hospitalization**:** HR = 1**.**96 **(**1**.**16–3**.**32**)** *P *= **.**01 Transition to HD: HR = 1.21 (0.48–3.02) *P *= 0.7 All-cause mortality: HR = 0.58 (0.26–1.31) *P *= .2 CV mortality: HR = 0.82 (0.31–2.16) *P *= .7
Yi *et al.* [24], 2018, China, Prospective, Single center	28.1 (15.5∼37.2)	784	48.8 ± 14.6	464/320	22.2 ± 3.3	CAPD (100%, *n* = 784)	30.7 (8.9–54.3)	72.70% (*n* = 570)	DM (20%) CV diseases (21.2%)	55.5% (*n* = 435)	Mandarin MoCA	MoCA score with variables Age: *r*’ = −.54, *P *< .001 Education level: *r*’ = .43, *P *< .001 Duration of dialysis, *r*’ = −.07, *P *= .04 BMI: *r*’ = −.09, *P *= .02 hs-CRP: *r*’ = −.21, *P *< .001 Serum albumin: *r*’ = 0.21, *P *< .001 Total cholesterol: *r*’ = −0.09, *P *= .01 Triglycerides: *r*’ = −0.09, *P *= .01 BUN: *r*’ = 0.08, *P *= .02 Serum creatinine: *r*’ = 0.24, *P *< .001	Risk of CI on mortality: HR = 1.18 (0.56–2.53) *P *= .66
Shea *et al.* [8], 2019, Hong Kong, Prospective, Single center	24	206	59 ± 13.7	112/94	N/A	CAPD (92.7%, *n* = 191) (92.7%) APD (7.3%, *n* = 15)	N/A	42.33% (*n* = 149)	DM (47.6%), HTN (90.3%), IHD (21.4%), PVD (14.1%), CVA (12.6%)	22.3% (*n* = 46)	HK-MoCA	Multivariable analyses for factors associated with CI: Age: adjusted OR 1.00, 95%CI 1.00–1.07, *P* = .03 Female: OR 3.57, 95% CI 1.60–7.97, *P* = .002 PVD: OR 3.46, 95% CI 1.33–9.01, *P* = .01 Hb level: OR 0.60, 95%CI 0.43–0.84, *P* = .003 Of note, this study reported % of helper assisted PD for 23.3% (*n* = 48)	CI-associated peritonitis (RR = 3.2, 95%CI 1.03–9.95; *P* = .04) Patients with CI vs non- CI PD-related peritonitis Incidence rate/year: 0.58 vs 0.42, *P *= .03 % suffered from peritonitis and exit-site infection: 26.1% vs 6.7%, *P* = .004 Unplanned hospitalization rate (episodes/year) 2.32 vs 1.11, *P *= .07 Number of ED admissions median (IQR) 1 (0–3) vs 1 (0–2), *P *= .03 Duration of admissions in days median (IQR) 4 (0–11) vs 1 (0–5), *P *= .04
Farragher *et al.* [23], 2019, Canada, Prospective, 3 dialysis centers	1	121	69.2 ± 10.1	81/40	N/A	N/A	N/A	41 (35%)	CAD (24%) PAD (13%) CVA (9%) DM (55%)	59% (*n* = 68/115)	MoCA ≤ 24	N/A	N/A
Huang *et al.* [9], 2021, China, Prospective, Single center	26.0 (13.5–35.6)	455	58.8 ± 10.8	303/152	22.8 (20.8–25.8)	CAPD (100%, *n* = 455)	29.1 (6.5–54.2)	78.24% (*n* = 356)	CV diseases (35.4%) CVA (19.3%) DM (38.4%) HTN (91.9%)	72.7% (*n* = 331)	MoCA	DM has OR 1.67;1.01–2.78, *P *= .045 for CI Subgroup of DM in PD to CI outcome - HbA1c OR 1.55; 1.01–2.36, *P *= .043 - CV disease OR, 2.93; 1.14–7.52, *P *= .026 - Rate of assisted PD in DM group = 39.4% vs non-DM group = 18.6%	Subgroup of DM in CAPD - CI was an independent risk factor for mortality adjusted HR, 7.22; 1.69–30.81, *P *= .008 Subgroup of non-DM in CAPD - Patients with CI had a higher risk of peritonitis than those without CI (0.20 vs. 0.09 episode/patient-year)

Abbreviations; 3MS, Modified Mini-Mental State Examination; TMT-B, Trail-Making Test B; HK-MoCA, Hong Kong Montreal Cognitive Assessment; d2-R, Test d2-Revision; ICD-9-CM, International Classification of Diseases, 9th revision, Clinical

### Methodological quality

The assessment of publication bias using the NOS on 12 cross-sectional studies found that six studies had a low risk of bias, with three studies classified as medium risk and another three studies classified as high risk. Only two out of seven cohort studies were classified as medium risk, while five of seven cohort studies were assessed as low risk of bias (Supplementary Tables S1 and S2).

### Prevalence of cognitive impairment in peritoneal dialysis patients

**Figure 2:**
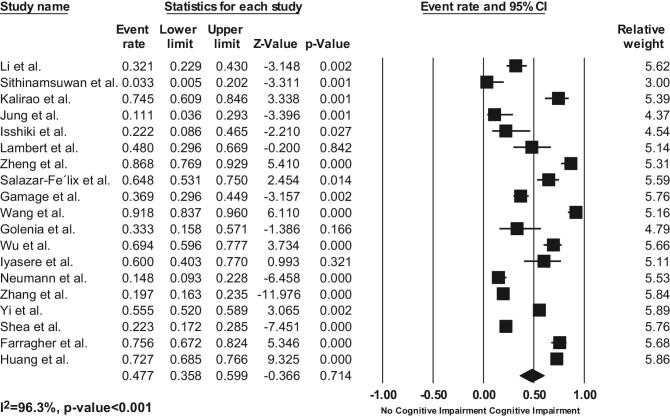
Forest plots of the pooled prevalence of CI in PD patients.

The pooled prevalence of CI in PD patients was 47.7% (95% CI: 35.8%–59.9%), defined by standardized cognitive exams. There was substantial heterogeneity among the studies (*I*^2^ = 96.3%, *P* < .001, Fig. 2). Each study may utilize more than one assessment tool. Of 19 studies, Montreal Cognitive Assessment (MoCA) was commonly used to identify CI by 10 studies, followed by Mental State Examination (MSE), which was employed by nine studies. Addenbrooke's Cognitive Examination (ACE) and Trail-Making Test (TMT) were used in two studies. The Repeatable Battery for the Assessment of Neuropsychological Status (RBANS), Test d2-Revision, and Subscale Cognitive Function from the Kidney Disease Quality of Life Short Form (KDQOL-CF) short form were performed in one study.

A study by Kalirao *et al.* was the only study that classified CI into different severities. The overall prevalence of CI in this study was 74.5%, divided into mild CI (7.8%), moderate CI (33.3%), and severe CI (33.3%). They performed a 45-minute battery of nine validated neuropsychological tests, including the Modified Mini-MSE and other standardized cognitive function tests, such as the Hopkins Verbal Learning Test-Revised, Color Trails 1 and 2 tests, and Stroop Interference test, to assess global cognitive function, memory, executive function, language, and visual-spatial domain. The severity of CI was classified by a score below the age-adjusted mean of each test and the number of impaired cognitive domains [15].

Subgroup analysis revealed that the prevalence of CI varied significantly across different types of cognitive examination (*P* for interaction <.001). The highest prevalence was observed with RBANS (pooled prevalence 74.5%; 95% CI 60.9%–84.6%; *I*² 0%, *P *= 1.00), followed by MoCA (pooled prevalence 64.6%; 95% CI 52.4%–75.2%; *I*² 95.05%, *P *< .001), ACE (pooled prevalence 36.5%; 95% CI 29.6%–44.1%; *I*² 0%, *P *= .77), MSE (pooled prevalence 20.2%; 95% CI 16.1%–25.1%; *I*² 78.55%, *P *< .001), and the lowest prevalence was found with TMT (pooled prevalence 18.8%; 95% CI 15.8%–22.3%; *I*² 24.98%, *P *= .248). Regarding the risk of bias of the studies, the pooled prevalence was as follows: 49.8% for those with a low risk of bias (95% CI 35.2%–64.5%; *I*² 97.13%, *P *< .001), 44.9% for those with a medium risk of bias (95% CI 18.2%–74.9%; *I*² 96.04%, *P *< .001), and 31.5% for those with a high risk of bias (95% CI 6.2%–76.2%; *I*² 92.9%, *P *< .001). In term of type of study designs, cross-sectional studies reported a higher prevalence of 49.8% (95% CI: 33.9–65.8%; *I*² 92.24%, *P *< .001), while cohort studies had a lower prevalence of 41.4% (95% CI: 24.5–60.5%; *I*² 98.24%, *P *< .001).

### Risk factors of cognitive impairment in peritoneal dialysis patients

#### Demographic characteristics

Demographic factors associated with CI are detailed in Table 1. Five of 19 studies [5, 8, 19, 24, 25] reported a consistent association between increased age and CI, with one of these studies [24] highlighting this factor as the strongest negative correlation [28]. Two studies [5, 8] found a positive relationship between female gender and CI. In addition, a lower year of leaving education was identified as a CI risk factor in five studies [5, 19, 21, 25, 24]. Based on limited evidence, higher BMI [24] and longer dialysis vintage [24] may be associated with increased CI risk.

#### Medical conditions

The associations between medical conditions and CI are summarized in Table 1. DM may increase the risk of CI [9], particularly when blood sugar levels are poorly controlled. Peripheral vascular disease [8] and lacunar infarction [18] have been examined consistently associated with cognitive decline. One study identified depression as a reversible risk factor for CI [25].

#### Laboratory factors

The association between CI and laboratory findings, including anemia [8], electrolyte disturbance (e.g. hyponatremia) [19], nutritional markers (e.g. serum creatinine [19, 24], serum albumin [24, 25], serum total cholesterol [24]), and inflammatory markers [e.g. high sensitivity C-reactive protein (hs-CRP)] [24], at diagnosis are summarized in Table 1. In brief, anemia, hyponatremia, and elevated hs-CRP were identified as significant risk factors for CI in PD patients, although the evidence is limited, as each factor was reported in only one study. Poor nutritional markers, such as hypoalbuminemia, low serum creatinine, and low total cholesterol, were found to have a negative correlation with CI.

### Clinical outcomes of cognitive impairment in peritoneal dialysis patients

Four longitudinal studies [8, 22, 24, 25] reported CI in PD patients was associated with clinical outcomes, including hospitalizations, peritonitis, exit-site infection, mortality, and quality of life.

#### Hospitalizations

PD patients with CI experienced a significantly 1.96-fold higher rate of hospitalization compared to those without CI (HR = 1.96, 95%CI 1.16–3.32; *P *= .01). A higher hospitalization rate (2.32 vs 1.11 episodes/year, *P *= .07), number of ED admission [median (IQR) 1 (0–3) vs 1 (0–2) days, *P *= .03], and duration of admission [median (IQR) 4 (0–11) vs 1 (0–5) days, *P *= .04] were found in the PD patients with CI group than in those without CI [8].

#### Peritonitis and exit-site infection

CI in the PD population was associated with a 3.2-fold increased incidence of peritonitis (RR = 3.2, 95%CI 1.03–9.95; *P *= .04). Among PD patients, those with CI had higher incidence of peritonitis than those without CI (0.58 vs 0.42 episode/year, *P *= .03 and 0.20 vs 0.09 episode/patient-year in the non-DM subgroup, respectively). Similar trends were reported on the incidence of peritonitis and exit-site infection (26.1% vs 6.7%, *P *= .004) [8].

#### Mortality

The overall impact of CI in PD on mortality was insignificant, except in the setting of DM subgroup. Huang *et al.* found CI to be an independent risk factor for mortality in diabetic CAPD patients (HR 7.22, 95%CI 1.69–30.81; *P *= .008) [9]. However, studies by Zhang *et al.* and Yi *et al.* did not find any significant relationship of CI in PD on all-cause mortality (HR = 0.58, 95%CI 0.26–1.31; *P *= .2 and HR = 1.18, 95% CI 0.56–2.53; *P *= .66, respectively) [24, 25].

#### Patient adherence and quality of life

Wu *et al.* compared CAPD patients between those with CI and without CI. They found that participants with CI had significantly lower compliance with diet fluid restriction, medication, and dialysis adherence (*P *< .05). CAPD patients with CI had lower QOL scores in physical function, general health, social function, emotional function, and mental health (*P *< .05) [22].

### Comparison between peritoneal dialysis and control groups

Eight studies [5–7, 15, 17, 19, 21, 26] compared baseline characteristics, prevalence of CI, and risk factors for CI between PD and control groups. None of these studies demonstrated CI-associated outcomes between PD and control groups.

#### Compared to non-CKD subjects

**Figure 3:**
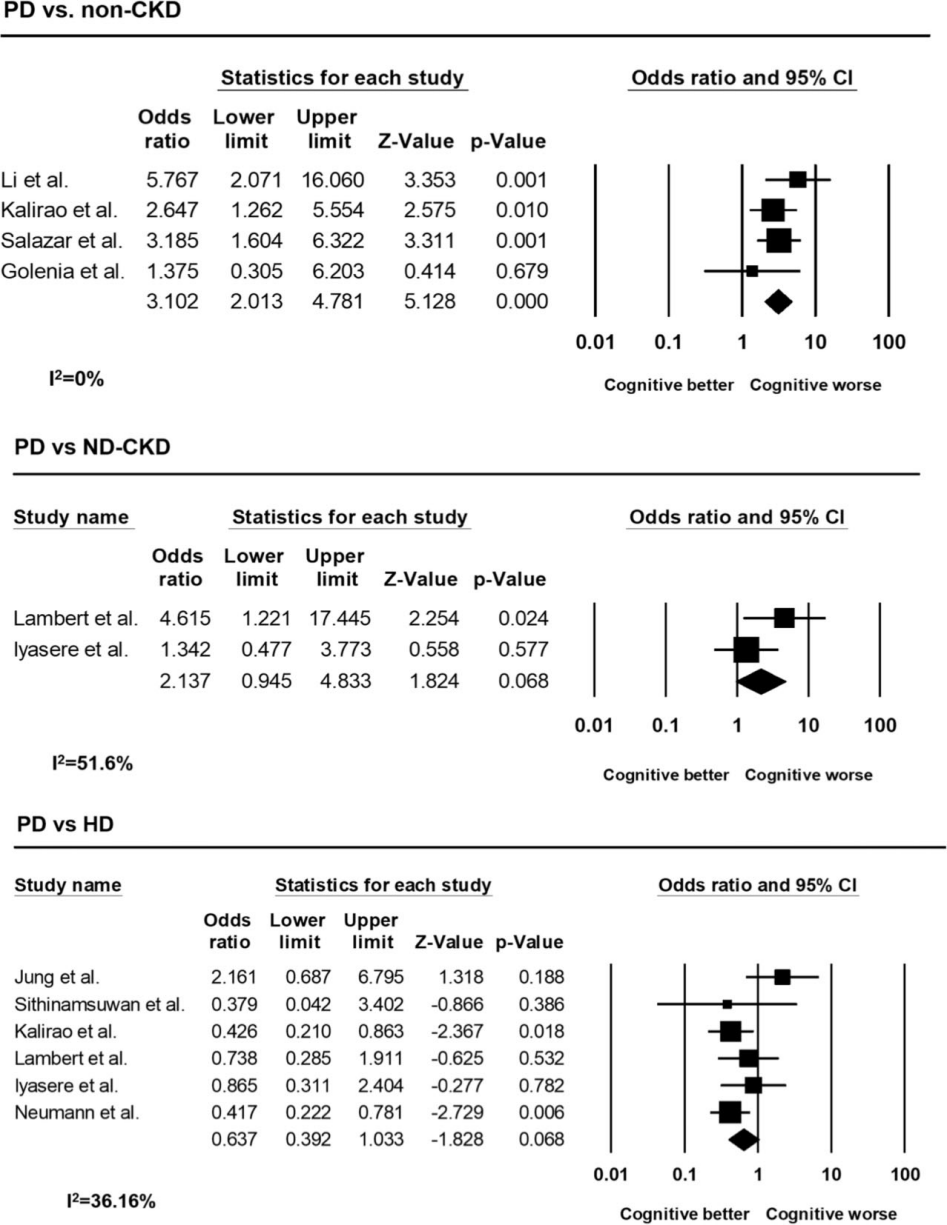
Comparison of the risk of CI between PD patients and control groups.

Compared to non-CKD particpants, four studies [5, 15, 19, 21], were included in the meta-analysis. The pooled analysis showed that participants with PD had a significantly higher risk of CI than those without CKD (OR 3.10, 95% CI 2.01–4.78; *P *< .001) (Fig. 3). There was no heterogeneity among the studies (*I*² 0%; *P *= .439). Kalirao *et al.* also classified CI into three different severities. The PD group had a higher proportion of moderate and severe CI than non-CKD group (33.3% vs 26.73% and 33.3% vs 12.87%, respectively) [15].

#### Compared to non-dialysis CKD

Two studies [17, 26] comparing non-dialysis CKD subjects to those with PD were included in the meta-analysis. The pooled analysis suggested that participants with PD demonstrated a trend toward higher risk of CI compared to those with non-dialysis CKD (OR 2.14, 95% CI 0.945–4.83; *P *= .068) (Fig. 3). Moderate heterogeneity was observed among the studies (*I*² 51.6%; *P *= .151). Similar trends were found when compared PD patients with non-dialysis CKD patients. Studies by Lambert *et al.* and Iyasere *et al.* found that PD patients had a higher prevalence of CI than CKD patients (48.0% vs 16.7% and 64.3% vs 58.3%, respectively) [17, 26].

#### Compared to hemodialysis

Six studies [7, 15, 6, 17, 26, 27] comparing HD subjects to those with PD were included in the meta-analysis. The pooled analysis indicated a trend toward a lower risk of CI for participants with PD compared to those undergoing HD (OR 0.64, 95% CI 0.39–1.03; *P *= .068) (Fig. 3). Mild heterogeneity was present among the studies (*I*² 36.16%; *P *= .166). According to the findings of Kalirao *et al.*, PD participants had a lower proportion of moderate and severe CI than HD subjects (33.3% vs 36.1% and 33.3% vs 27.3%, respectively). Compared to the control group, the risk of moderate to severe CI was 2.58 times higher in the PD group (OR = 2.58, 95%CI 1.02–6.53), while it was 3.16 times higher in the HD group (OR = 3.16, 95%CI 1.91–5.24) [15].

#### Compared to renal transplantation

PD patients tended to have a higher prevalence of CI than renal transplant patients. Lambert *et al.* compared prevalence of CI in PD and transplant patients (48.0% vs 19.2%) [17].

### Meta-regression

**Figure 4:**
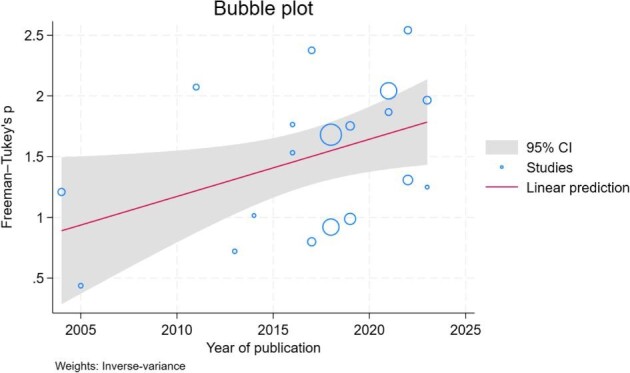
Meta-regression between year of publication and prevalence of CI in PD patients.

Meta-regression analysis showed that the prevalence of CI was significantly associated with the publication year of the included studies (*β* = 0.047; *P* = .03; *R*^2 ^= 9.81%) (Figure 4)**.** However, the analysis revealed that the prevalence of CI was not influenced by age (*P* = .925) and dialysis vintage (*P* = .879) (Supplementary Figures S1 and S2).

### Assessment of publication bias

The funnel plot and Egger's regression model demonstrated an absence of publication bias for the prevalence of CI (*P *= .44 by the Egger test) (Supplementary Figure S3) in the studies included in the meta-analysis.

## DISCUSSION

Our systematic review investigated the prevalence, risk factors, and clinical outcomes of CI in PD patients. A high prevalence of CI was observed, with a pooled estimate prevalence of 47.7%. The most common standardized cognitive assessment tools used in the included studies were MoCA and MSE. PD patients had a higher prevalence of CI compared to non-CKD, non-dialysis CKD, and renal transplant individuals, while their prevalence of CI was lower than in HD patients.

PD patients with CI experience significantly poorer clinical outcomes compared to those without CI, including increased hospitalization rates, higher incidences of peritonitis and exit-site infections, reduced treatment adherence, and poorer quality of life. While the impact on mortality remains inconclusive, there is some evidence suggesting a potential link in diabetic PD patients. Our findings highlight the critical need for regular cognitive screening in PD patients, particularly among older individuals, females, and those with lower education levels. Clinicians should be aware of the high prevalence of CI in this population, as it significantly increases the risk of hospitalization and peritonitis. By identifying at-risk patients early, healthcare providers can implement timely interventions, such as tailored education, enhanced monitoring, and supportive care, to mitigate these adverse outcomes. These findings underline the importance of integrating cognitive assessments into routine PD care protocols.

Our findings demonstrate a higher pooled prevalence of CI compared to a study by Shea *et al.* (47.7% vs. 28.7%) [29]. This difference could be attributed to several factors, which were increased sample size and variation in cognitive assessment tools. Our review included a larger number of studies (19 vs. 8) and patients (2882 vs. 1736), leading to greater statistical power to detect a significant difference in prevalence. The studies included in Shea *et al.*’s review primarily used the MSE, while over half of the studies in our review utilized the MoCA. MoCA is considered more sensitive in detecting mild CI. However, our systematic review findings were consistent with the findings of Shea *et al.* [29] in identifying risk factors for CI, including older age, female sex, lower education, and hyponatremia. Both studies demonstrate the association between CI and a higher rate of hospitalization and peritonitis.

The prevalence of CI varied across different cognitive tests, potentially due to differences in accuracy of diagnostic tools [30, 31]. This variation can cause substantial heterogeneity in the pooled prevalence. For example, MoCA has demonstrated a higher sensitivity than MMSE to identify early CI [32, 33], while ACE III yield a higher sensitivity and specificity than MoCA and MMSE [34]. Our meta-regression analysis indicated that the year of publication is significantly related to the prevalence of CI. This trend might be attributed to the change in use of cognitive exams. Specifically, enrolled studies published between 2004 and 2014 predominantly used MSE, while those from 2016 to 2023 mainly used MoCA. Given MoCA has a higher sensitivity than MSE to identify early CI [32, 33], the prevalence of CI in the recent studies were technically higher than the older ones.

Several factors were reviewed in this study as potential risks of CI in PD. PD itself, older age, female gender, and lower levels of education, were associated with increased prevalence of CI. Anemia, hyponatremia, poor nutritional markers, and elevated inflammatory marker at diagnosis, were associated with poorer cognitive test scores. Dialysis-related factors contributing to CI included chronic inflammation, uremic toxin accumulation, and vitamin D deficiency. Dialysis provokes a pro-inflammatory state. Observational studies have linked elevated levels of inflammatory markers with CI in PD patients [35, 36]. Uremic toxins can traverse the blood–brain barrier, leading to neurodegeneration [37]. Vitamin D status was associated with cognitive function among PD patients [37].

The aging process, accompanied by cellular senescence of neurons and microglia, results in micro-inflammation, directly causing memory decline [38]. Studies in CKD did not find any significant sex difference on prevalence of CI [39]. Only two studies in our results reported a significant risk of being female on CI in PD [5, 8]. Further research is necessary clarify the role of sex in cognitively impaired PD patients. Anemia, hyponatremia, and markers of malnutrition were also related with CI in PD. Anemia impairs oxygen delivery to the brain, affecting brain metabolism and exacerbating CI [40]. Abnormal nutritional markers indicated that malnutrition play a role in CI among PD patients [41].

Although our analysis shows a trend toward a lower risk of CI in PD patients compared to those on hemodialysis, this finding did not reach statistical significance. Nevertheless, the trend aligns with previous studies [7, 15, 6, 17, 26, 27] suggesting that the more stable hemodynamic environment in PD, compared to the hemodynamic fluctuations and cerebral hypoperfusion associated with HD, may contribute to better cognitive outcomes [42]. Beyond shared common risk factors, HD patients are susceptible to intradialytic cerebral hypoperfusion [43], hemodynamic fluctuations [44], and arterial stiffness [45], which can lead to cerebral ischemic injury to vulnerable vascular beds [46]. Conversely, PD provides a consistent hemodynamic environment [42]. Future studies with larger sample sizes are warranted to further explore this relationship and determine whether PD might offer protective effects against CI.

Early management of risk factors may help delay the onset of CI in PD patients, improving their overall outcomes. Further research is needed to establish standardized cognitive assessment tools and guidelines for the timely identification and management of CI in PD patients.

### Strengths

We present the most up-to-date data on CI in PD patients. This study has a larger sample size compared to the sample size of the previous meta-analysis [29], as mentioned earlier. The shift toward using the MoCA, a more sensitive cognitive assessment tool, contributed to the higher CI prevalence observed in this study compared to the previous estimate. In addition, we comprehensively examined and reported common risk factors and clinical outcomes in PD population.

### Limitations

Several limitations should be noted. First, our findings were affected by significant heterogeneity, partly due to differences in diagnostic tests used across studies, which led to variability in the reported pooled prevalence of CI. The varying cut-off points for CI diagnosis among studies further complicated the interpretation. Additionally, differences in education levels among study populations, presented in various formats, contributed to the heterogeneity. Despite performing subgroup analyses, the heterogeneity was not completely resolved, possibly due to considerable differences in the methodologies, populations, and other unmeasured factors across the studies. Future research should aim to standardize cognitive assessment methods and study designs to reduce heterogeneity and improve the comparability of results. Another limitation of this study is the reliance on observational studies, which are inherently prone to selection and confounding biases. Although we utilized the NOS to assess the quality of the included studies, the potential for bias cannot be entirely mitigated. Observational designs lack the controlled environment of randomized studies, which may affect the internal validity of the findings and limit our ability to infer causality. Additionally, we excluded studies published in languages other than English, which could have introduced language bias. While this decision was made to ensure consistency and allow for accurate interpretation, we acknowledge that relevant data published in other languages might have been omitted, potentially affecting the comprehensiveness of our analysis. Future research should consider including non-English studies to minimize this bias and provide a more global perspective on CI in PD patients. Last, relevant factors contributing to CI, including BMI, smoking habits, alcohol use, level of assistance, income, dialysis adequacy, and type of PD, were not consistently collected across studies.

## CONCLUSION

CI is common in PD patients, with a pooled prevalence rate of 47.7%. Reported major risk factors for CI included female gender, older age, and a lower level of education. CI is linked to increased rates of hospitalization and peritonitis. Our systematic review and meta-analysis provide a comprehensive understanding of CI in PD patients, an underexplored but clinically important population. The high prevalence of CI and its association with adverse outcomes such as hospitalization and peritonitis underscore the need for routine cognitive assessments in PD care. By identifying patients at higher risk—especially those who are older, female, or have lower levels of education—clinicians can intervene early to potentially improve patient outcomes. These findings can inform future clinical guidelines and support the development of targeted interventions aimed at reducing the burden of CI in this vulnerable population.

## Supplementary Material

sfae312_Supplemental_Files

## Data Availability

The data underlying this article are available in the article and in its online supplementary material.

## References

[1] Thurlow JS, Joshi M, Yan G et al. Global epidemiology of end-stage kidney disease and disparities in kidney replacement therapy. Am J Nephrol 2021;52:98–107. 10.1159/000514550 33752206 PMC8057343

[2] Chuasuwan A, Pooripussarakul S, Thakkinstian A et al. Comparisons of quality of life between patients underwent peritoneal dialysis and hemodialysis: a systematic review and meta-analysis. Health Qual Life Outcomes 2020;18:191. 10.1186/s12955-020-01449-2 32552800 PMC7302145

[3] Gupta R, Woo K, Yi JA. Epidemiology of end-stage kidney disease. Semin Vasc Surg 2021;34:71–78. 10.1053/j.semvascsurg.2021.02.010 33757639 PMC8177747

[4] United States Renal Data System . 2022 USRDS Annual Data Report: Epidemiology of kidney disease in the United States. 2022; National Institutes of Health, National Institute of Diabetes and Digestive and Kidney Diseases, Bethesda, MD, USA.

[5] Li JSC . Prevalence of cognitive impairment among elderly Chinese continuous ambulatory peritoneal dialysis patients. Hong Kong J Nephrol 2004;6:22–30. 10.1016/S1561-5413(09)60122-8

[6] Jung S, Lee YK, Choi SR et al. Relationship between cognitive impairment and depression in dialysis patients. Yonsei Med J 2013;54:1447–53. 10.3349/ymj.2013.54.6.1447 24142650 PMC3809877

[7] Sithinamsuwan P, Niyasom S, Nidhinandana S et al. Dementia and depression in end stage renal disease: comparison between hemodialysis and continuous ambulatory peritoneal dialysis. J Med Assoc Thai 2005;88:S141–S147. 16858952

[8] Shea YF, Lee MC, Mok MM et al. Self-care peritoneal dialysis patients with cognitive impairment have a higher risk of peritonitis in the second year. Perit Dial Int 2019;39:51–58. 10.3747/pdi.2018.00048 30087176

[9] Huang X, Yi C, Wu M et al. Risk factors and clinical outcomes of cognitive impairment in diabetic patients undergoing peritoneal dialysis. Kidney Blood Press Res 2021;46:531–40. 10.1159/000514172 34229326

[10] Wang Y, Zhang HX, Wang YC et al. A survey of cognitive function in peritoneal dialysis patients. Ther Apher Dial 2022;26:822–6. 10.1111/1744-9987.13777 34898008

[11] Moher D, Liberati A, Tetzlaff J et al. Preferred reporting items for systematic reviews and meta-analyses: the PRISMA statement. PLoS Med 2009;6:e1000097. 10.1371/journal.pmed.1000097 19621072 PMC2707599

[12] von Elm E, Altman DG, Egger M et al. The Strengthening the Reporting of Observational Studies in Epidemiology (STROBE) statement: guidelines for reporting observational studies. J Clin Epidemiol 2008;61:344–9. 10.1016/j.jclinepi.2007.11.008 18313558

[13] Wells GA, 'Connell SBOD, Peterson J et al. The Newcastle-Ottawa Scale (NOS) for assessing the quality of nonrandomised studies in meta-analyses. http://www.ohri.ca/programs/clinical_epidemiology/oxford.asp (15 June 2024, date last accessed).

[14] Herzog R, Alvarez-Pasquin MJ, Diaz C et al. Are healthcare workers' intentions to vaccinate related to their knowledge, beliefs and attitudes? A systematic review. BMC Public Health 2013;13:154. 10.1186/1471-2458-13-154 23421987 PMC3602084

[15] Kalirao P, Pederson S, Foley RN et al. Cognitive impairment in peritoneal dialysis patients. Am J Kidney Dis 2011;57:612–20. 10.1053/j.ajkd.2010.11.026 21295896 PMC3121243

[16] Isshiki R, Kobayashi S, Iwagami M et al. Cerebral blood flow in patients with peritoneal dialysis by an easy Z-score imaging system for brain perfusion single-photon emission tomography. Ther Apher Dial 2014;18:291–6. 10.1111/1744-9987.12107 24965295

[17] Lambert K, Mullan J, Mansfield K et al. Comparison of the extent and pattern of cognitive impairment among predialysis, dialysis and transplant patients: a cross-sectional study from Australia. Nephrology 2017;22:899–906. 10.1111/nep.12892 27505310

[18] Zheng K, Wang H, Hou B et al. Malnutrition-inflammation is a risk factor for cerebral small vessel diseases and cognitive decline in peritoneal dialysis patients: a cross-sectional observational study. BMC Nephrol 2017;18:366. 10.1186/s12882-017-0777-1 29262796 PMC5738894

[19] Salazar-Felix NA, Martin-Del-Campo F, Cueto-Manzano AM et al. Prevalence of mild cognitive impairment in automated peritoneal dialysis patients. Nephrol Dial Transplant 2021;36:2106–11. 10.1093/ndt/gfab238 34375410

[20] Gamage I, Dhar A, Tregaskis P et al. Frequency and risk factors for cognitive dysfunction in peritoneal dialysis patients. Nephrology 2022;27:945–52. 10.1111/nep.14117 36190395

[21] Golenia A, Zolek N, Olejnik P et al. Prevalence of cognitive impairment in peritoneal dialysis patients and associated factors. Kidney Blood Press Res 2023;48:202–8. 10.1159/000530168 36940679 PMC10124757

[22] Wu C, Yu R, Li Q et al. Exploring the impact of cognitive impairments on treatment compliance and quality of life in patients with Continuous Ambulatory Peritoneal Dialysis (CAPD). Medicine 2023;102:e35813. 10.1097/MD.0000000000035813 37904409 PMC10615453

[23] Farragher JF, Oliver MJ, Jain AK et al. PD assistance and relationship to co-existing geriatric syndromes in incident peritoneal dialysis therapy patients. Perit Dial Int 2019;39:375–81. 10.3747/pdi.2018.00189 31123074

[24] Yi C, Lin J, Cao P et al. Prevalence and prognosis of coexisting frailty and cognitive impairment in patients on continuous ambulatory peritoneal dialysis. Sci Rep 2018;8:17305. 10.1038/s41598-018-35548-4 30470776 PMC6251896

[25] Zhang YH, Yang ZK, Wang JW et al. Cognitive changes in peritoneal dialysis patients: a multicenter prospective cohort study. Am J Kidney Dis 2018;72:691–700. 10.1053/j.ajkd.2018.04.020 30007504

[26] Iyasere O, Okai D, Brown E. Cognitive function and advanced kidney disease: longitudinal trends and impact on decision-making. Clin Kidney J 2017;10:89–94. 10.1093/ckj/sfw128 28638609 PMC5469575

[27] Neumann D, Mau W, Wienke A et al. Peritoneal dialysis is associated with better cognitive function than hemodialysis over a one-year course. Kidney Int 2018;93:430–8. 10.1016/j.kint.2017.07.022 29042081

[28] Ciesielska N, Sokolowski R, Mazur E et al. Is the Montreal Cognitive Assessment (MoCA) test better suited than the Mini-Mental State Examination (MMSE) in mild cognitive impairment (MCI) detection among people aged over 60? Meta-analysis. Psychiatr Pol 2016;50:1039–52. 10.12740/PP/45368 27992895

[29] Shea YF, Lee MC, Mok MM et al. Prevalence of cognitive impairment among peritoneal dialysis patients: a systematic review and meta-analysis. Clin Exp Nephrol 2019;23:1221–34. 10.1007/s10157-019-01762-1 31250148

[30] Zhou H, Sabbagh M, Wyman R et al. Instrumented trail-making task to differentiate persons with no cognitive impairment, amnestic mild cognitive impairment, and alzheimer disease: a proof of concept study. Gerontology 2017;63:189–200. 10.1159/000452309 27855415 PMC5311006

[31] Senda M, Terada S, Takenoshita S et al. Diagnostic utility of the Addenbrooke's Cognitive Examination—III (ACE-III), Mini-ACE, Mini-Mental State Examination, Montreal Cognitive Assessment, and Hasegawa Dementia Scale-Revised for detecting mild cognitive impairment and dementia. Psychogeriatrics 2020;20:156–62. 10.1111/psyg.12480 31448862

[32] Zadikoff C, Fox SH, Tang-Wai DF et al. A comparison of the mini mental state exam to the Montreal Cognitive Assessment in identifying cognitive deficits in Parkinson's disease. Mov Disord 2008;23:297–9. 10.1002/mds.21837 18044697

[33] Videnovic A, Bernard B, Fan W et al. The Montreal Cognitive Assessment as a screening tool for cognitive dysfunction in Huntington's disease. Mov Disord 2010;25:401–4. 10.1002/mds.22748 20108371

[34] Li X, Yang L, Yin J et al. Validation study of the Chinese version of Addenbrooke's Cognitive Examination III for Diagnosing Mild Cognitive Impairment and Mild Dementia. J Clin Neurol 2019;15:313–20. 10.3988/jcn.2019.15.3.313 31286702 PMC6620441

[35] Kurella Tamura M, Tam K, Vittinghoff E et al. Inflammatory markers and risk for cognitive decline in chronic kidney disease: the CRIC study. Kidney International Reports 2017;2:192–200. 10.1016/j.ekir.2016.10.007 28439566 PMC5399682

[36] Crowe K, Quinn TJ, Mark PB et al. “Is it removed during dialysis?”—Cognitive dysfunction in advanced kidney failure—a review article. Front. Neurol 2021;12:1–18. 10.3389/fneur.2021.787370 PMC8674209 34925220

[37] Liu G-L, Pi H-C, Hao L et al. Vitamin D status is an independent risk factor for global cognitive impairment in peritoneal dialysis patients. PLoS ONE 2015;10:e0143782. 10.1371/journal.pone.0143782 26630385 PMC4668056

[38] Raz N, Daugherty AM. Pathways to brain aging and their modifiers: free-radical-induced energetic and neural decline in senescence (FRIENDS) model—a mini-review. Gerontology 2018;64:49–57. 10.1159/000479508 28858861 PMC5828941

[39] Wang XH, He Y, Zhou H et al. Risk factors for cognitive impairment in patients with chronic kidney disease. World J Psychiatry 2024;14:308–14. 10.5498/wjp.v14.i2.308 38464766 PMC10921279

[40] Cheng BC, Chen PC, Chen PC et al. Decreased cerebral blood flow and improved cognitive function in patients with end-stage renal disease after peritoneal dialysis: an arterial spin-labelling study. Eur Radiol 2019;29:1415–24. 10.1007/s00330-018-5675-9 30105409 PMC6510858

[41] Daradkeh G, Essa MM, Al-Adawi SS et al. Nutritional status and cognitive impairment in elderly. Pakistan J Biol Sci 2014;17:1098–105. 10.3923/pjbs.2014.1098.1105 26027153

[42] Van Biesen W, Pletinck A, Verbeke F et al. Acute central hemodynamic effects of peritoneal dialysis. Contrib Nephrol 2009;163:96–101. 10.1159/000223786 19494601

[43] Wolfgram DF . Intradialytic cerebral hypoperfusion as mechanism for cognitive impairment in patients on hemodialysis. JASN 2019;30:2052–8. 10.1681/ASN.2019050461 31511363 PMC6830804

[44] Lin YK, Kao CC, Tseng CH et al. Noninvasive hemodynamic profiles during hemodialysis in patients with and without heart failure. Cardiorenal Med 2020;10:243–56. 10.1159/000506470 32268337

[45] Angermann S, Baumann M, Wassertheurer S et al. Pulse wave velocity is associated with cognitive impairment in hemodialysis patients. Clin Sci 2017;131:1483–93. 10.1042/CS20170087 28495909

[46] Findlay MD, Dawson J, Dickie DA et al. Investigating the relationship between cerebral blood flow and cognitive function in hemodialysis patients. JASN 2019;30:147–58. 10.1681/ASN.2018050462 30530658 PMC6317612

