# Supplementary material for: Addressing cognitive impairment in peritoneal dialysis: a systematic review and meta-analysis of prevalence, risk factors, and outcomes
Source: Clin Kidney J. 2024 Oct 15;17(11):sfae312. doi: 10.1093/ckj/sfae312 (PMC11565236; doi:10.1093/ckj/sfae312)
Supplement: sfae312_Supplemental_Files [file sfae312_supplemental_files.zip › Supplementary1 CI in PD 091524.docx]

**Supplemental Material 1**

***Supplementary tables: Risk of bias assessment***

**Table S1. Risk of bias assessment using the Newcastle-Ottawa Scale adapted for cross-sectional studies**

|  | Cross-sectional studies | | | |
| --- | --- | --- | --- | --- |
| Study | Selection  (maximum 3 points) | Comparability  (maximum 2 points) | Outcome  (maximum 2 points) | Quality |
| Li et al. [5] | *** | ** | ** | 7  (low risk of bias) |
| Sithinamsuwan et al. [7] | *** | * | * | 5  (medium risk of bias) |
| Kalirao et al. [15] | *** | * | ** | 6  (low risk of bias) |
| Jung et al. [6] | *** | - | * | 4  (high risk of bias) |
| Isshiki et al. [16] | ** | * | * | 4  (high risk of bias) |
| Lambert et al. [17] | *** | * | * | 5  (medium risk of bias) |
| Zheng et al. [18] | ** | ** | ** | 6  (low risk of bias) |
| Salazar-Fe´lix et al. [19] | *** | ** | ** | 7  (low risk of bias) |
| Gamage et al. [20] | ** | ** | ** | 6  (low risk of bias) |
| Wang et al. [10] | *** | * | * | 5  (medium risk of bias) |
| Golenia et al. [21] | *** | ** | ** | 7  (low risk of bias) |
| Wu et al. [22] | ** | * | * | 4  (high risk of bias) |

**Table S2. Risk of bias assessment using the Newcastle-Ottawa scale for observational studies**

|  | Cohort Studies | | | |
| --- | --- | --- | --- | --- |
| Study | Selection  (maximum 4 points) | Comparability  (maximum 2 points) | Outcome  (maximum 3 points) | Quality |
| Iyasere et al. [26] | **** | ** | ** | 8  (low risk of bias) |
| Neumann et al. [27] | **** | ** | *** | 9  (low risk of bias) |
| Zhang et al. [25] | *** | ** | *** | 8  (low risk of bias) |
| Yi et al. [24] | *** | ** | *** | 8  (low risk of bias) |
| Shea et al. [8] | *** | * | *** | 7  (medium risk of bias) |
| Farragher et al. [23] | *** | ** | ** | 7  (medium risk of bias) |
| Huang et al. [9] | *** | ** | *** | 8  (low risk of bias) |

***Supplementary Figures***

**Figure S1. Meta-regression models between prevalence of cognitive impairment in peritoneal dialysis patients and age
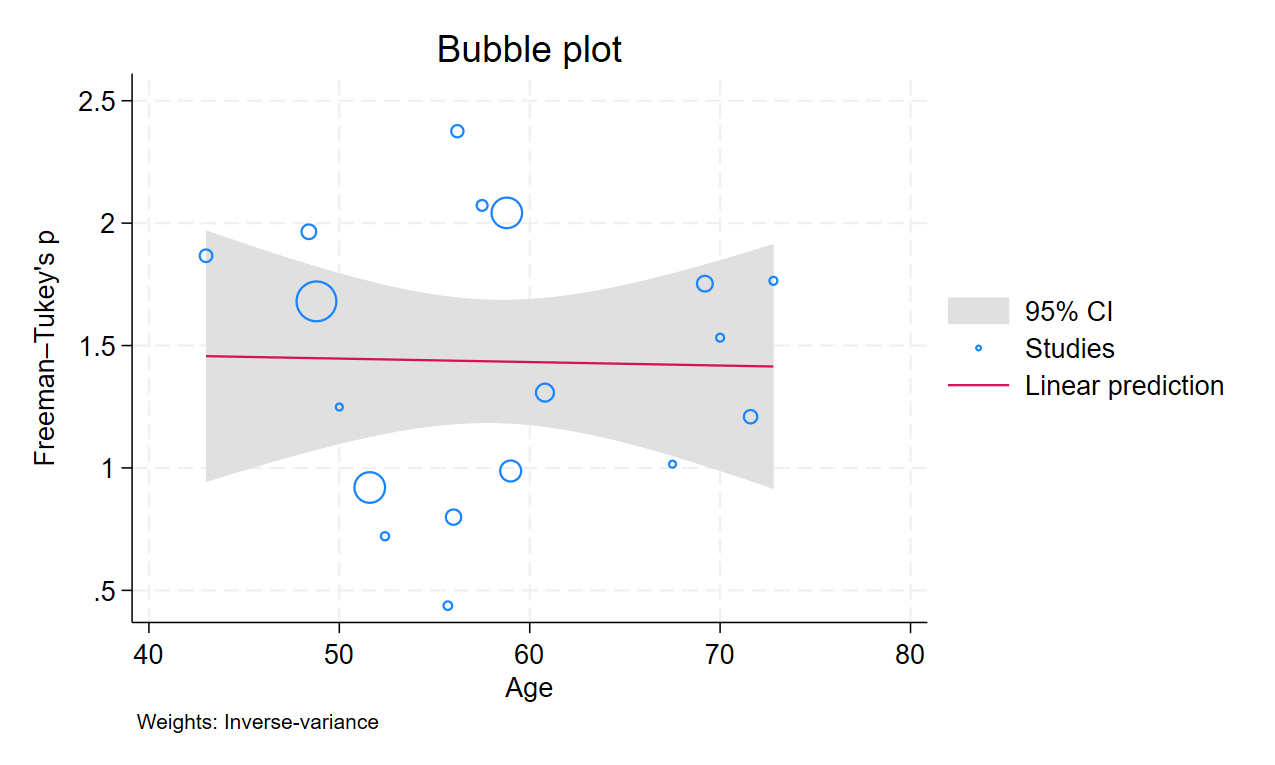
**

*^Meta-regression analysis showed that the prevalence of CI was not associated with age (p-value = 0.925).^*

**Figure S2. Meta-regression models between prevalence of cognitive impairment in peritoneal dialysis patients and duration of dialysis**

**
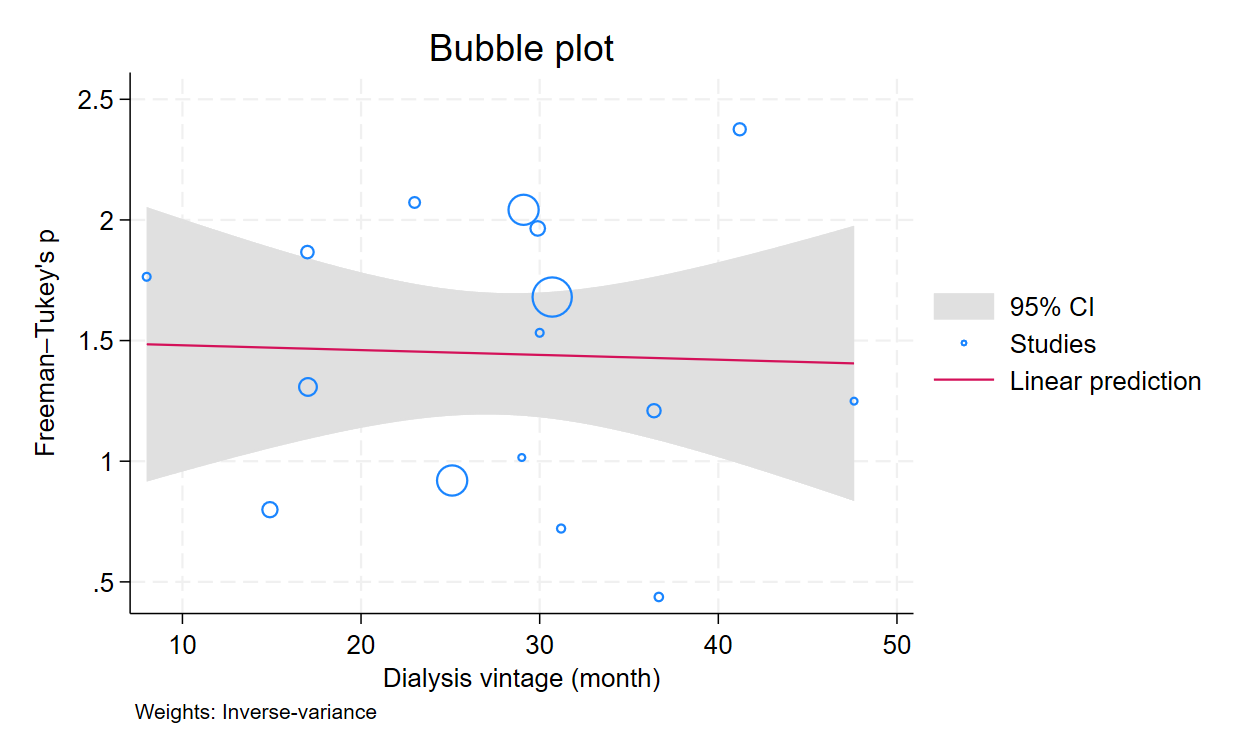
**

*^Meta-regression analysis showed that the prevalence of CI was not associated with dialysis vintage (p-value = 0.879).^*

**Figure S3. Funnel plot for *publication bias assessment***

***
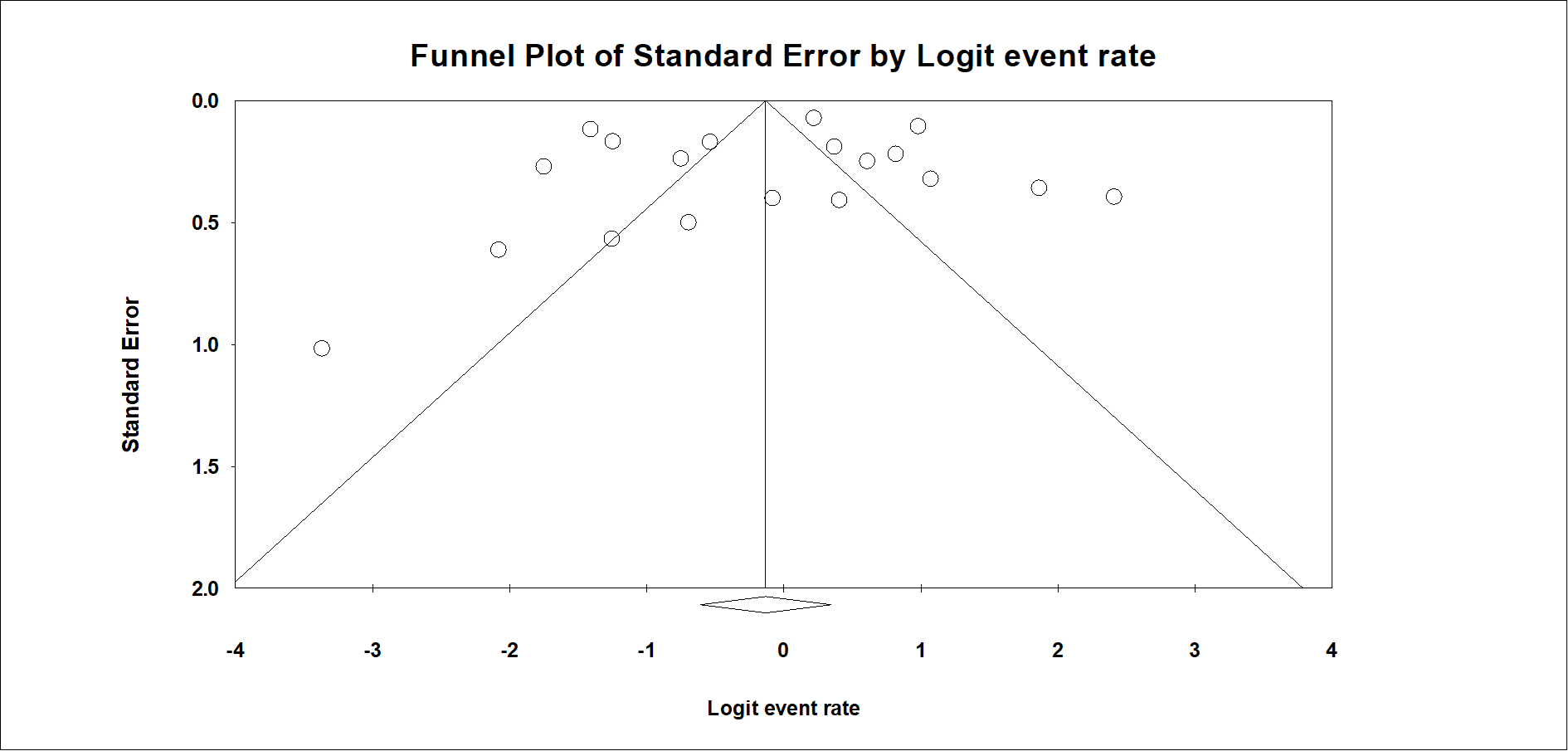
***

*^Funnel plot and Egger’s regression model demonstrated an absence of publication bias for the prevalence of CI (p=0.44) in the studies included in the meta-analysis.^*
