# Supplementary figures and images for: Addressing cognitive impairment in peritoneal dialysis: a systematic review and meta-analysis of prevalence, risk factors, and outcomes
Source: Clin Kidney J. 2024 Oct 15;17(11):sfae312. doi: 10.1093/ckj/sfae312 (PMC11565236; doi:10.1093/ckj/sfae312)

Bubble plot

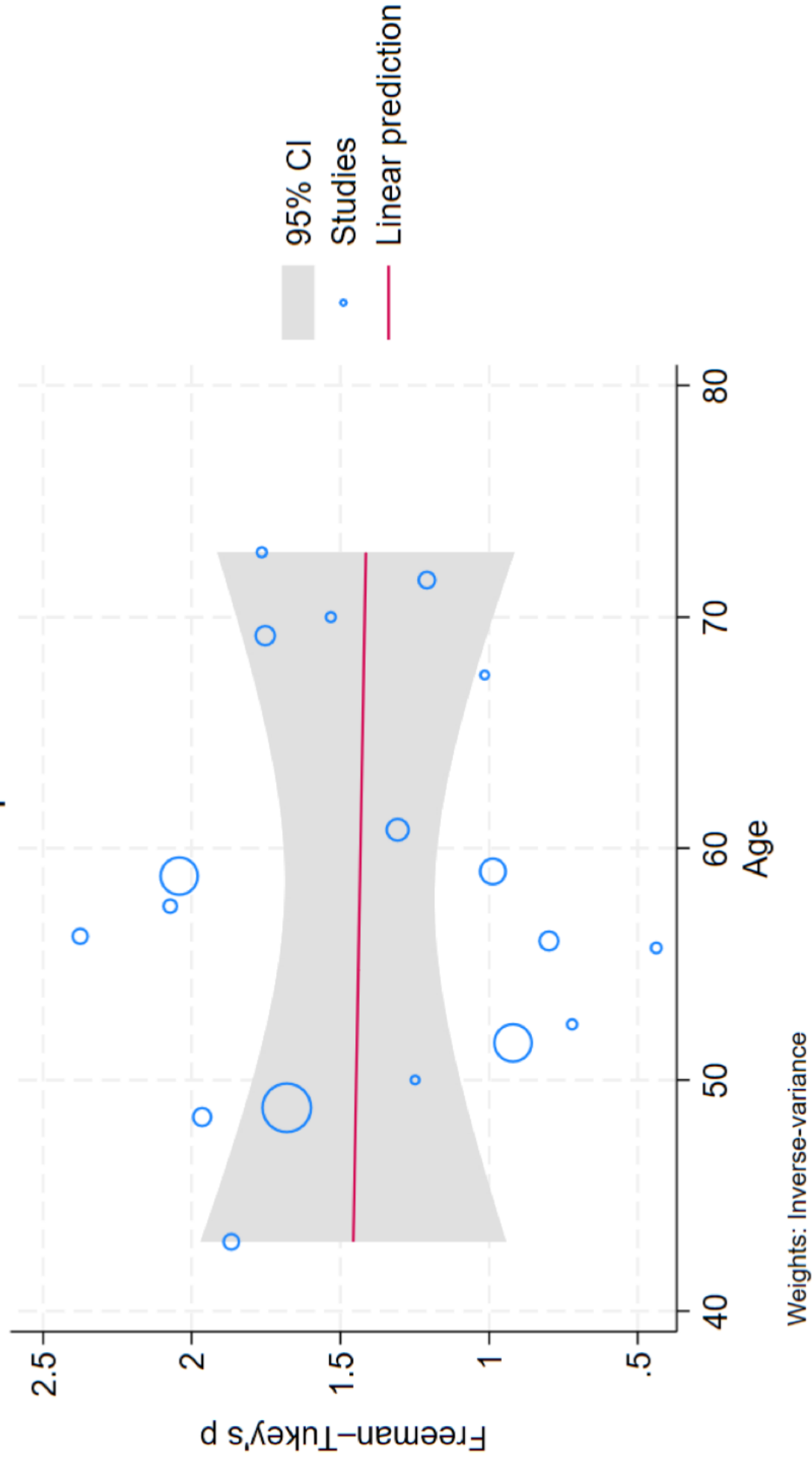

Supplement: sfae312_Supplemental_Files [file sfae312_supplemental_files.zip › Figure S1 CI in PD.pdf]

Bubble plot

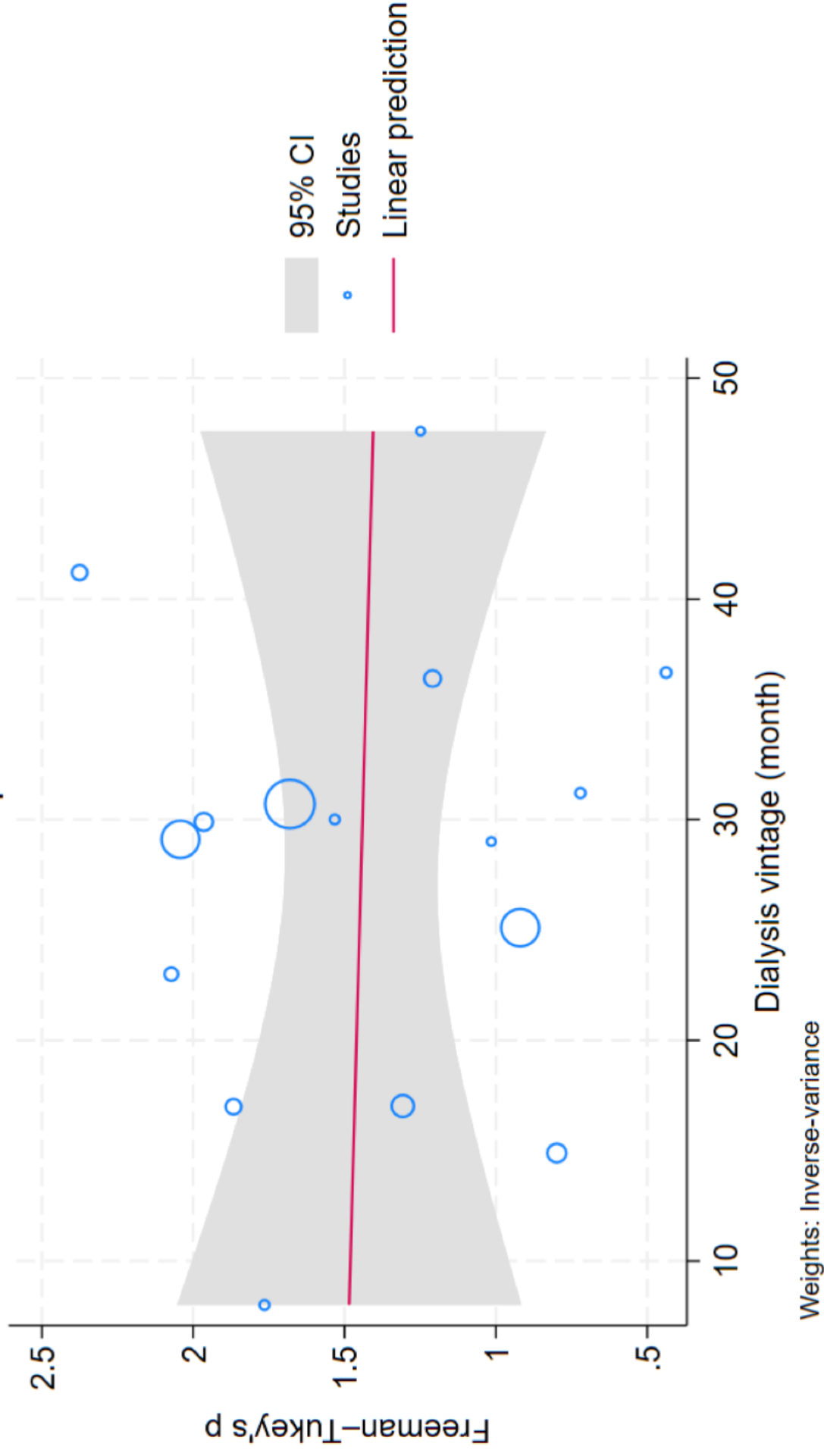

Supplement: sfae312_Supplemental_Files [file sfae312_supplemental_files.zip › Figure S2 CI in PD.pdf]

Funnel Plot of Standard Error by Logit event rate

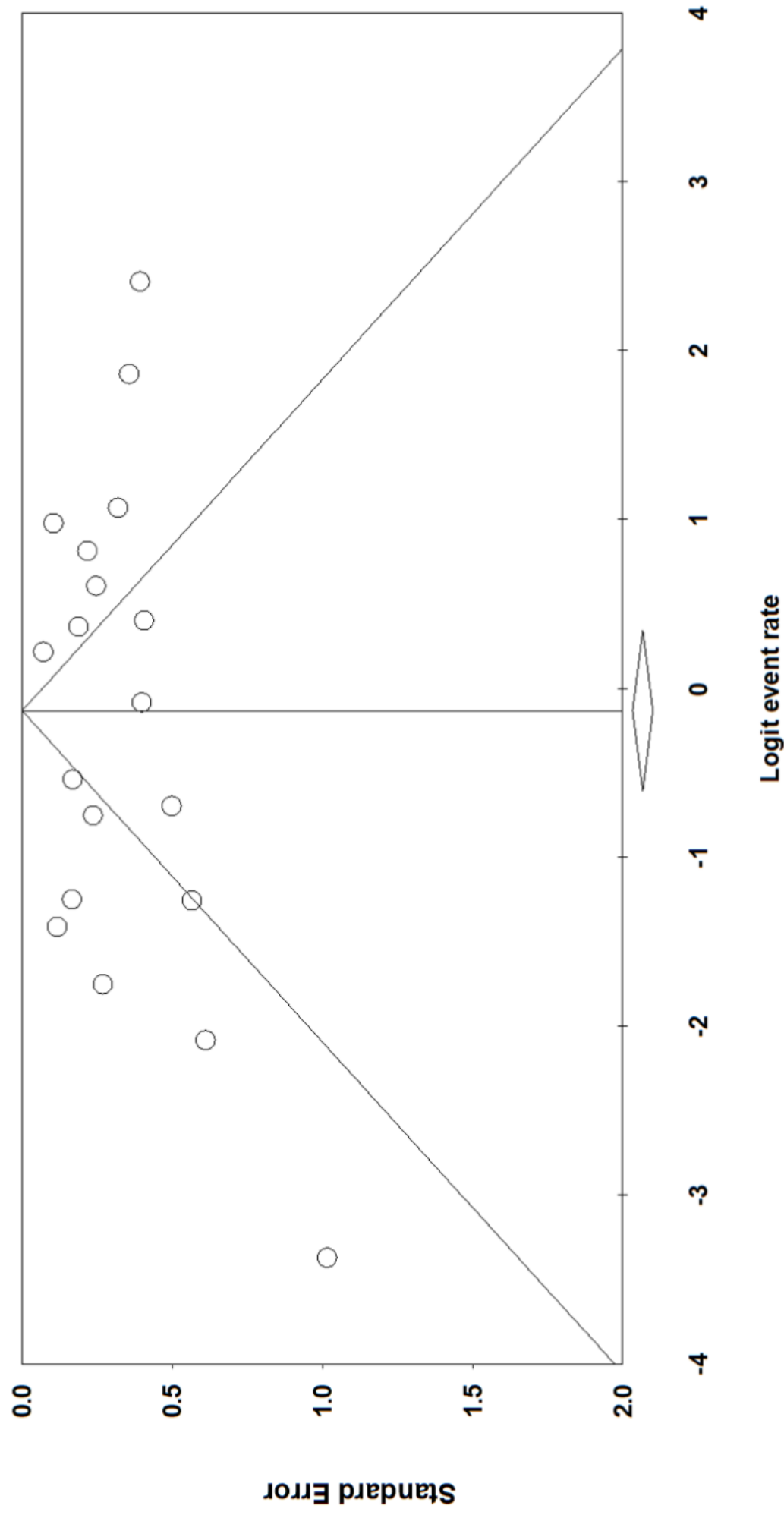

Supplement: sfae312_Supplemental_Files [file sfae312_supplemental_files.zip › Figure S3 CI in PD.pdf]
